# Attributing practice variation by its sources: the case of varicose veins treatments in the Netherlands

**DOI:** 10.1186/s12913-023-10328-7

**Published:** 2023-11-30

**Authors:** Luca Schippa, Katalin Gaspar, Eric van der Hijden, Xander Koolman

**Affiliations:** 1 Intelligence to Integrity (i2i), Amsterdam, The Netherlands; 2 School of Business and Economics, Talma Institute / VU University Amsterdam, Section Health Economics, Amsterdam, The Netherlands; 3 CPB Netherlands Bureau for Economic Policy Analysis, The Hague, The Netherlands; 4 Zilveren Kruis (Achmea), Amersfoort, The Netherlands

**Keywords:** Health care, Hospital, Primary care, Unwarranted practice variation, I11, I13, I18

## Abstract

**Background:**

Unwarranted practice variation refers to regional differences in treatments that are not driven by patients’ medical needs or preferences. Although it is the subject of numerous studies, most research focuses on variation at the end stage of treatment, i.e. the stage of the treating specialist, disregarding variation stemming from other sources (e.g. patient preferences, general practitioner referral patterns). In the present paper, we introduce a method that allows us to measure regional variation at different stages of the patient journey leading up to treatment.

**Methods:**

A series of logit regressions estimating the probability of (1) initial visit with the physician and (2) treatment correcting for patient needs and patient preferences. Calculating the coefficient of variation (CVU) at each stage of the patient journey.

**Results:**

Our findings show large regional variations in the probability of receiving an initial visit, The CVU, or the measure of dispersion, in the regional probability of an initial visit with a specialist was significantly larger (0.87–0.96) than at the point of treatment both conditional (0.14–0.25) and unconditional on an initial visit (0.65–0.74), suggesting that practice variation was present before the patient reached the specialist.

**Conclusions:**

We present a new approach to attribute practice variation to different stages in the patient journey. We demonstrate our method using the clinically-relevant segment of varicose veins treatments. Our findings demonstrate that irrespective of the gatekeeping role of general practitioners (GPs), a large share of practice variation in the treatment of varicose veins is attributable to regional variation in primary care referrals. Contrary to expectation, specialists’ decisions meaningfully diminish rather than increase the amount of regional variation.

## Introduction

When medical decisions are based on guidelines, we expect clinically comparable patients to receive comparable treatments. However, health services research has shown that clinical practice is highly variable across regions, and this variation is often unrelated to differences in population need [1]. This phenomenon is referred to as unwarranted “medical practice variation”.

Unwarranted practice variation in a health care system is an indicator for substandard quality of care and for inefficiencies and inequities: the identification of its sources is important for policy makers, professionals, and insurers alike. Existing measures of practice variation primarily rely on data gathered on treated patients at the final stage of the patient journey, generally treatment performed by a specialist. This method neglects to take into account other potential sources of variation, such initial contacts in primary care and/or initial patient preferences regarding seeking treatment. Therefore, it likely overestimates the magnitude of variation caused by the specialist as well as discounts the responsibility of the general practitioners (GPs) in weeding out patients without legitimate medical needs. GPs represent the first point of contact in health care in the Netherlands. All Dutch residents are required by law to register at one of several hundred private GP offices and obtain a GP’s referral for any initial visit with a specialist. Once a GP referral is obtained, the patient visits the specialist for an initial consult at which point the specialist determines whether and what type of treatment is necessary.

In the present study we propose a new method that allows for the comparison of the magnitude of regional variation in treatments, by looking at two different stages of the patient journey: (1) regional variation measured at the point of the initial visit with the specialist, and (2) at the point of treatment. We take the treatment of varicose veins as an example to illustrate our method.

Varicose veins are dilated superficial veins in the lower extremities [2]. Although in majority of the cases it is asymptomatic, sometimes pressure and pain in the leg, and occasionally a rupture of the vein, can occur. Hence, varicose veins treatments are often considered an elective procedure where patient demand can be highly sensitive to patient characteristics (e.g. being female, having high socio-economic status (SES)) [3]. Research from the United Kingdom has shown large regional variation not only in the volumes of varicose veins operations but also in volume of referrals by primary care trusts [4, 5]. This suggests that at least some of the variation may be present prior to reaching the treating physician’s clinic.

The outline of our paper is as follows. In Sect. 1, we introduce the current methodology used to assess practice variation, we provide a brief overview on the treatment of varicose veins and on Dutch health care system. In Sect. 2, we describe our data and present our proposed methods. In Sect. 3, we present our results, we discuss our findings and conclude.

### Overview of practice variation literature

Research on practice variations dates back to 1937 when J Alison Glover registered an unexplained variation in tonsillectomy-rates among British school children [6]. But it is John E. Wennberg of the Dartmouth Institute who is most often referred to as the pioneer of the subject [7]. Researchers since then have established the presence of unwarranted variation at different levels of aggregation: among hospitals and/or groups of doctors, among individual doctors of similar expertise, and between small geographic regions (i.e. small area variations) [7, 8]. Some of the disparities in treatments may exist as a result of differences in patient needs (i.e. warranted variation), while other variation could signal unmet health care needs, or that health care resources are being used inappropriately (i.e. unwarranted variation). Demonstrating the need to understand and tackle *unwarranted* practice variation, Wennberg states that *“physicians in some hospital markets practice medicine in ways that have extremely adverse implications for the cost of care, motivated perhaps by reasons of their own or their patients' convenience”,* and that *“substantial cost savings and improvements in quality could be realized without fear that needed services were being withheld”* [9]*.*

Existing methods for identifying geographical practice variation either rely on the calculation of incidence or prevalence after correcting for differences in patient populations [10], or use hierarchical or random effect models that allow for the existence of some provider-specific ‘random variation’ [11]. One study has addressed the relationship between geographical practice variation and GP referrals by identifying the impact of having access to a GP on hospital admissions [12]. This study, however, investigates referrals from multiple small areas to a *single* general hospital, therefore it does not address variation *between* providers.

### Overview of varicose veins

Varicose veins are dilated superficial veins in the lower extremities that are very common between the ages of 40 and 80 [2]. They are typically asymptomatic, but in certain cases they may cause a sense of fullness, pressure, and pain or hyperesthesia in the legs. In extreme cases, varicose veins may rupture and bleed [13]. In the Netherlands, treatment of varicose veins is only covered by basic insurance if the case classification is at least 3 in a 0 to 6 scale, but since this classification is based on patient reporting (of pain) and physician observation, grading is open to interpretation. Therefore, isolating cases where treatment is medically necessary from treatments that are solely based on the patient’s preference, can be difficult.

#### Identifying cases of medical necessity vs. preference

Factors associated with higher probability of treatment are *age, female sex, obesity, previous pregnancies, history of deep vein thrombosis,* and *height * [14]*.* However, some factors (e.g. being female, relatively high socio-economic status *(SES)*) could be positively correlated with patients’ propensity to seek medical help, thereby over-representing certain types of clients in the treated population. There is no evidence in the literature that *income*, *profession* and *SES* would influence the risk of developing painful varicose veins [15, 16]. Studies come to different conclusions regarding the relevance of *sex * [15]*.* Treatment for varicose veins is more frequent in women than in men, but this could also occur because women are more likely to seek medical help related to varicose veins for aesthetic reasons. Furthermore, studies based on a comprehensive physical examination found *“no significant difference in the overall prevalence of hyphenweb or reticular varices between the sexes”* [16]*.*

### Background to the Dutch health care system

The Dutch health care system is a heavily regulated market-oriented system with mandatory universal health insurance. Plan-holders are reimbursed fully for services provided within the basic package on top of the annually determined compulsory front-end deducible (€385 in 2019). The primary care sector has an important gatekeeping function: patients must first visit their GPs with their concerns and must obtain a referral for a medical specialist consult based on medical—rather than esthetic—needs in order to get their health care costs reimbursed by their insurance [17]. Patients who do not follow this path will not show up in claims data.

## Methods and data

### Data sources

Our claims dataset was provided by Zilveren Kruis (ZK), the largest health insurer in the Netherlands, representing ~ 5.1 million clients or 30.4% of Dutch population in 2017 (Intelligence, 2018). The dataset comprises plan holders’ records including an anonymous individual identification code, age, sex, 2 digit postal code and various other plan-holder information for the year 2017 (see Table 1 for list of all variables). We identified claims registered in the specialties of dermatology (310) and surgery (303) with the diagnosis “superficial vein pathology/varicose veins” as treated.

**Table 1:** List of variables

Variable	Source	Description	Variable type	Needs (N)/Preferences (P)
Region	ZK	Identifies the 2-digit postal code where the patient is registered	Categorical (90 categories)	
Age	ZK	Age of the client in 2017	Categorical (4 categories)	N
Sex	ZK		Categorical (2 categories)	P
Obesity	ZK	“True” if the client received medical help for obesity between 2015 and 2017	Categorical (2 categories)	N
Childbirth	ZK	“True” if the client had a birth in the last between 2015 and 2017	Categorical (2 categories)	P
Average SES category per postal code	CBS	Data available per postal code, used as a proxy for the client’s economic status	Categorical (3 categories- Low/Medium/High)	P
Average income category per postal code	CBS	Data available per postal code, used as a proxy for the client’s economic status	Categorical (3 categories- Low/Medium/High)	P
Urbanization level per postal code	CBS	Urban density per postal code	Categorical (4 categories)	P
Initial visit with specialist for varicose veins	ZK	Dependent variable: “true” if the client had any claim for varicose veins in 2017	Categorical (2 categories)	
Treatment of varicose veins	ZK	Dependent variable: “true” if the client had a claim for treatment of varicose veins in 2017	Categorical (2 categories)	

Footnote: *N* stands for patient medical needs, *P* stands for patient preferences

Data from the Dutch Central Bureau of Statistics (CBS) and from the Social Cultural Planning Bureau (SCP) were used to enrich our dataset with small-area characteristics related to the plan-holder (e.g. SES and the rate of urbanization).

### Attribution method 

#### Definition of outcome variables and hypothesis

Most practice variation studies focus on the probability of treatment at the end-stage of the treatment process relative to the entire population of policy-holders. (1) This is the starting point of our analysis. In the present paper we disentangle the sources of this variation. Therefore, we calculate the (2) probability of the initial consult, which allows us to quantify the variation that originated from the process leading up to the consult to overall regional practice variation. This contains the contribution of the gatekeeping GPs, and unmeasured differences in need and preferences. Moreover, we present (3) the probability of treatment conditional on an initial visit that allows the study of the contribution of clinics that perform the treatment. We assume treatment performing clinics exert little influence on the initial visit.

Hence, we begin by modeling three probabilities for each policy-holder starting with the treatment that we aim to attribute to the preceding steps:

- $Pr\left(T\right)$: probability of receiving treatment.
- $Pr\left(I\right)$: probability of an initial consult.
- $Pr\left(T|I\right)$: probability of receiving treatment conditional on having an initial consult.

where T stands for treatment and I stands for initial consult.

Our goal is to calculate the probabilities defined above and to estimate a measure of regional variation. For this purpose we use the coefficient of variation (CVU), i.e. the measure of dispersion in the calculated probabilities, between postal codes.^1^

As per Diehr (1992), our formula for calculating the CVU for $Pr\left(T\right)$ and $Pr\left(I\right)$ is:

1a $$\overline{CVU }\left(Pr\left(T\right)\right)=\frac{{SD}_{T}}{{\overline{\mathrm{X}} }_{T}}$$

1b $$\overline{CVU }\left(Pr\left(I\right)\right)=\frac{{SD}_{I}}{{\overline{\mathrm{X}} }_{I}}$$

where ${SD}_{T} ({SD}_{I})$ stands for the standard deviation of the average probability of having a treatment (an initial visit) with a specialist and ${\overline{\mathrm{X}} }_{T}$ (${\overline{\mathrm{X}} }_{I}$) for the average probability of treatment (an initial visit) between all postal codes. Similarly, we calculate the CVU for $Pr\left(T|I\right)$ as:

2 $$\overline{CVU }\left(Pr\left(T|I\right)\right)=\frac{{SD}_{T|I}}{{\overline{X} }_{T|I}}$$

Formally our hypotheses becomes:

3a $$\begin{array}{ccc}\mathrm{Hypothesis }\ 1:& {H}_{0}:\overline{CVU }\left(Pr(T)\right)=& \overline{CVU }\left(Pr\left(I\right)\right)\end{array}$$

3b $$\begin{array}{ccc}\mathrm{Hypothesis }\ 2:& {H}_{0}:\overline{CVU }\left(Pr(I)\right)=& \overline{CVU }\left(Pr\left(I\right)\right)\end{array}$$

3c $$\begin{array}{ccc}\mathrm{Hypothesis }\ 3:& {H}_{0}:\overline{CVU }\left(Pr(T)\right)=& \overline{CVU }\left(Pr\left(I\right)\right)\end{array}$$

Hypothesis 1 represents the overall regional dispersion between treatment and initial visits without identifying how this dispersion is affected by the decision made before the initial visit and the decisions of the specialist. Hypothesis 3 tests how the regional dispersion of treatment given the dispersion stemming from the initial visit compares to the overall regional dispersion of treatment. That is, how does the decision of the specialist to treat alter the overall dispersion of treatment. Hypothesis 2 evaluates a similar setup, but instead compares the dispersion of treatments conditional on initial visit to overall treatment, it compares to the dispersion at the initial state (i.e. at the initial visit).

If Hypothesis 1 cannot be rejected and assuming both terms are positive, then we can conclude that regional variation in treatment is not different from regional variation in the initial visit that follows from the referral. It indicates that specialists do not significantly alter this regional variation, that is, the specialists’ decision does not lead to more appropriate care. If Hypothesis 2 cannot be rejected and both terms are positive, then we can conclude that the magnitude of variation stemming from the specialist *conditional on* prior events is not significantly different from the magnitude of variation originating from prior events. This arises if both steps in the patient journey contribute equally to the practice variation in treatment. If Hypothesis 3 cannot be rejected and both terms are positive, then we cannot rule out that all of the practice variation in treatment arises from the decisions made at the specialist's center following the initial visit that resulted from the referral, and all the variation observed may stem from the specialist. We use a bootstrap method (Efron & Tibshirani, 1986) to calculate standard errors around our estimated CVUs. This method entails resampling our dataset 10,000 times and calculating probabilities, as well as appropriate CVUs for each sample.^2^

### Correcting for patient needs and patient preferences

We use a series of logistic regressions to calculate the above probabilities. In Model 1, we begin by calculating crude (unadjusted) probabilities without any corrections.^3^ Model 2, we correct for patient needs and in Model 3 we correct for patient needs and preferences.

Formally, we calculate probabilities for each individual as:

4a $$Pr\left({T}_{i}|{D}_{a},\dots ,{D}_{A}\right)=\frac{{e}^{w}}{1+{e}^{w}}$$

4b $$Pr\left({I}_{i}|{D}_{a},\dots ,{D}_{A}\right)=\frac{{e}^{w}}{1+{e}^{w}}$$

5 $${{w}_{1}= \beta }_{0}+{\beta }_{a}{D}_{a}+\dots +{\beta }_{A}{D}_{A}+{e}_{i}\ for\ Model\ 1$$

6 $${{w}_{2}= \beta }_{0}+{\beta }_{a}{D}_{a}+\dots +{\beta }_{A}{D}_{A}+\theta {X}_{i}+{e}_{i}\ for\ Model\ 2$$

7 $${{w}_{3}= \beta }_{0}+{\beta }_{a}{D}_{a}+\dots +{\beta }_{A}{D}_{A}+\theta {X}_{i}+\gamma {Z}_{i}+{e}_{i}\ for\ Model\ 3$$

where i is an individual in region a, ${D}_{a}$ to ${D}_{A}$ are dummy variables for each region, ${X}_{i}$ stands for all relevant variables indicating patient needs and Z_i_ stands for patient preferences (see Table 1 for full list of variables). Our coefficients of interest will be ${\beta }_{a}$ to ${\beta }_{A}$, where ${\beta }_{a}$ represents the difference in needs- and preference-adjusted probability in region *a* compared to the reference group.

We proceed by estimating similar regressions for $Pr\left({T}_{i}|{I}_{i}, {D}_{A}\right)$ 

8 $$Pr\left({T}_{i}|{{I}_{i}, D}_{a},\dots ,{D}_{A}\right)=\frac{{e}^{w}}{1+{e}^{w}}$$

where

9 $${{w}_{1}= \beta }_{0}+{\beta }_{a}{D}_{a}+\dots +{\beta }_{A}{D}_{A}+{\pi R}_{i}+{e}_{i}\ for\ Model\ 1$$

10 $${{w}_{2}= \beta }_{0}+{\beta }_{a}{D}_{a}+\dots +{\beta }_{A}{D}_{A}+\theta {X}_{i}+{\pi R}_{i}+{e}_{i}\ for\ Model\ 2$$

11 $${{w}_{3}= \beta }_{0}+{\beta }_{a}{D}_{a}+\dots +{\beta }_{A}{D}_{A}+\theta {X}_{i}+\gamma {Z}_{i}+{\pi R}_{i}+{e}_{i}\ for\ Model\ 3$$

Predicted values are then calculated for individual *i* and region *a* as:

12 $$\widehat{Pr}\left({I}_{i}|{D}_{a},\dots ,{D}_{A}\right)=\frac{{e}^{w}}{1+{e}^{w}}$$

where

13 $${{w}_{1}= \widehat{\beta }}_{0}+{\widehat{\beta }}_{a}{D}_{a}+\dots +{\widehat{\beta }}_{A}{D}_{A}+{e}_{i}\ for\ Model\ 1$$

14 $${w}_{2}={\widehat{\beta }}_{0}+ {\widehat{\beta }}_{a}{D}_{a}+\dots +{\widehat{\beta }}_{A}{D}_{A}+\widehat{\theta }\overline{{X }_{i}}+{e}_{i}\ for\ Model\ 2$$

15 $${{w}_{3}= \widehat{\beta }}_{0}+{\widehat{\beta }}_{a}{D}_{a}+\dots +{\widehat{\beta }}_{A}{D}_{A}+\widehat{\theta }\overline{{X }_{i}}+\widehat{\gamma }\overline{{Z }_{i}}+{e}_{i}\ for\ Model\ 3$$

where $\overline{X}$ and $\overline{\mathrm{Z}}$ stands for patient-needs and preferences variables fixed at one category in order to eliminate their effect from the regression. (see Table 3). And similarly

16 $$\widehat{Pr}\left({T}_{i}|{D}_{a},\dots ,{D}_{A}, {I}_{i}\right)=\frac{{e}^{w}}{1+{e}^{w}}$$

where

17 $${{w}_{1}= \widehat{\beta }}_{0}+{\widehat{\beta }}_{a}{D}_{a}+\dots +{\widehat{\beta }}_{A}{D}_{A}+\widehat{\pi }{I}_{i}+ {e}_{i}\ for\ Model\ 1$$

18 $${w}_{2}={\widehat{\beta }}_{0}+ {\widehat{\beta }}_{a}{D}_{a}+\dots +{\widehat{\beta }}_{A}{D}_{A}+\widehat{\theta }\overline{{X }_{i}}+\widehat{\pi }{I}_{i}+{e}_{i}\ for\ Model\ 2$$

19 $${{w}_{3}= \widehat{\beta }}_{0}+{\widehat{\beta }}_{a}{D}_{a}+\dots +{\widehat{\beta }}_{A}{D}_{A}+\widehat{\theta }\overline{{X }_{i}}+\widehat{\gamma }\overline{{Z }_{i}}+\widehat{\pi }{I}_{i}+{e}_{i}\ for\ Model\ 3$$

We sum up the predicted probabilities for all individuals to obtain the needs-adjusted probabilities per postal code.

Next, we calculate the CVU for $Pr\left(T\right)$, $Pr\left(I\right)$ and $Pr\left(T|I\right)$ based on Eqs. (1a, 1b) and (2). We bootstrap our results to obtain the standard deviation.

## Results

### Descriptive statistics and regression model selection

In Table 2 we present the descriptive statistics for all variables used. In our dataset, 27,067 of the nearly 5.1 million plan holders were seen for an initial visit for varicose veins. In total, 20,453 plan holders received treatment. Majority of the those with only an initial visit were between 41 and 60 years old (51% of the total sample), but the share of this age-group was considerably lower (41%) among those that ended up with a treatment. Contrary to the medical literature which points to a positive correlation between age and the need for treatment, patients in higher age groups (61–70 and 70 +) represented only a fraction of the treated population (11% of the total sample was between 61 and 70 and 9.5% was 70 or above). Majority of the patients seen for only an initial visit were female (85% of the total), but this share was lower for treated patients (60% of the treated sample). The share of patients with obesity and recent childbirth were insignificant among those that received treatment: approx. 0.00% in our dataset had a recent medical claim indicating obesity and 0.01% had recent childbirth. On the other hand, the level of urbanization indicated that those from the least urbanized environments were the most likely to receive an initial visit and eventually treatment (37.8% and 33.5% of the total sample, respectively).

**Table 2:** Descriptive statistics

	**Initial visit only**	**Treated**	**No visit, not treated**	**Overall**
	**(*N =* 6,614)**	**(*N =* 20,453)**	**(*N =* 5,129,907)**	**(*N =* 5,156,974)**
**Age categories**				
0–40	828 (12.5%)	7,683 (37.6%)	2,518,263 (49.1%)	2,526,774 (49.0%)
41–60	3,375 (51.0%)	8,593 (42.0%)	1,404,164 (27.4%)	1,416,132 (27.5%)
61–70	1,153 (17.4%)	2,242 (11.0%)	569,852 (11.1%)	573,247 (11.1%)
70 +	1,258 (19.0%)	1,935 (9.5%)	637,628 (12.4%)	640,821 (12.4%)
**Female**				
Mean	0.85	0.60	0.50	0.50
**Obese**				
Mean	0.00	0.00	0.00	0.00
**Childbirth**				
Mean	0.01	0.01	0.02	0.02
**Level of urbanization**				
Quartile 1	2,498 (37.8%)	6,858 (33.5%)	1,279,888 (24.9%)	1,289,244 (25.0%)
Quartile 2	1,612 (24.4%)	5,423 (26.5%)	1,282,209 (25.0%)	1,289,244 (25.0%)
Quartile 3	1,580 (23.9%)	5,119 (25.0%)	1,282,544 (25.0%)	1289,243 (25.0%)
Quartile 4	924 (14.0%)	3,053 (14.9%)	1285,266 (25.1%)	1,289,243 (25.0%)

For some treated patients (5345 patients) no initial visit claim could be found. The most probable explanation is that the initial visit for these patients occurred before 2017 and it was not included in our dataset. For the purpose of Pr(T), Pr(T|I), we assumed that these patients received an initial visit. However, we did not include them when estimating Pr(I).

### Regression output

In Table 3, we present the marginal effects for Pr(T), Pr(I) and Pr(T|I) regressions described in Sect. 2.3. The marginal effects in regressions 1–3 are considerably smaller when compared to regressions 4–6, as a consequence of the large and heterogeneous dataset used for those regressions, whereas regressions 4–6 build on a smaller and less heterogeneous group (i.e. all policy-holders vs. policy-holders with an initial visit).

**Table 3:** Logit regression output – Marginal Effects

	Pr(T)			Pr(I)			Pr(T|I)		
	(1)	(2)	(3)	(4)	(5)	(6)	(7)	(8)	(9)
	Model 1	Model 2	Model 3	Model 1	Model 2	Model 3	Model 1	Model 2	Model 3
obesitas		− 0.0021	− 0.0020		− 0.0003	− 2e − 04		0.1925	0.1775
		(0.0049)	(0.0047)		(0.0038)	(0.0028)		(0.1391)	(0.1279)
childbirth		− 0.0013	− 0.0014		− 0.0002	− 2e − 04		− 0.1011 +	− 0.0202
		(0.0030)	(0.0033)		(0.0023)	(0.0022)		(0.0609)	(0.0312)
age_cat4160		0.0020	0.0019		0.0011	8e − 04		− 0.2166 *	− 0.1963 +
		(0.0047)	(0.0044)		(0.0135)	(0.0099)		(0.1091)	(0.1031)
age_cat6170		0.0009	0.0009		0.0012	9e − 04		− 0.3202 **	− 0.3047 **
		(0.0022)	(0.0020)		(0.0142)	(0.0102)		(0.1041)	(0.1083)
age_cat70		0.0000	− 0.0001		0.0009	6e − 04		− 0.3792 ***	− 0.3510 **
		(0.0002)	(0.0004)		(0.0107)	(0.0068)		(0.1043)	(0.1110)
incomelow			0.0006			1e − 04			0.0007
			(0.0014)			(0.0008)			(0.0099)
incomemiddle			0.0007			0			0.0148
			(0.0016)			(0.0004)			(0.0122)
seslow			− 0.0005			0			− 0.0185
			(0.0012)			(0.0001)			(0.0130)
sesmiddle			− 0.0004			0			− 0.0060
			(0.0010)			(0.0003)			(0.0074)
female			0.0011			4e − 04			− 0.1687 +
			(0.0025)			(0.0046)			(0.1006)
urban2			− 0.0002			− 1e − 04			0.0081
			(0.0005)			(0.0008)			(0.0100)
urban3			0.0002			0			0.0082
			(0.0006)			(0.0000)			(0.0116)
urban4			− 0.0008			− 1e − 04			0.0132
			(0.0018)			(0.0008)			(0.0150)
overweight_middle					0.0000	0		0.0476 +	0.0501
					(0.0002)	(0.0001)		(0.0285)	(0.0306)
overweight_high					− 0.0001	0		0.0529	0.0516
					(0.0009)	(0.0006)		(0.0330)	(0.0328)
Num.Obs	515,696	515,696	515,696	515,696	515,696	515,696	27,067	27,067	27,067
AIC	26,300.8	26,063.4	25,909.7	15,359.9	14,690.5	14,195.6	29,410.4	27,652.0	26,298.6
Wald-test p-value	0.00	0.00	0.00	0.00	0.00	0.00	0.00	0.00	0.00

Footnote: Regional dummy variables omitted from Table 3. Hence, Model 1 for all three groups of regressions has no presented variables

In regressions 1–6, the marginal effects of all patient needs and preferences variables are small in magnitude and often insignificant. This indicates that most of the variation within regions cannot be directly explained by these factors and there is a risk that potentially important confounding variables were not controlled for. Adding variables for patient needs and preferences improves the model fit based on Akaike Information Criterion (AIC). Model 3, which includes both need and preference variables, indicated the best model fit.

In regression 8–9, the predictive power of having received treatment for obesity in the two years prior to 2017 is strong and positive (approx. 19 and 18% increase in probability). Similarly, with recent childbirth: the marginal effects on receiving treatment are strong and negative (-10.2 and -0.02%). The probability of treatment given an initial visit is largest for the youngest age-group (up to 40 years of age). This is demonstrated by the large and negative marginal effects on all three other age-categories. Income, SES and the level of urbanization are only weak predictors of receiving treatment given an initial visit. On the other hand, being female indicates a statistically weak, albeit in magnitude large (-16.9%) effect in regression 9.

### Hypothesis testing

**Fig. 1:**
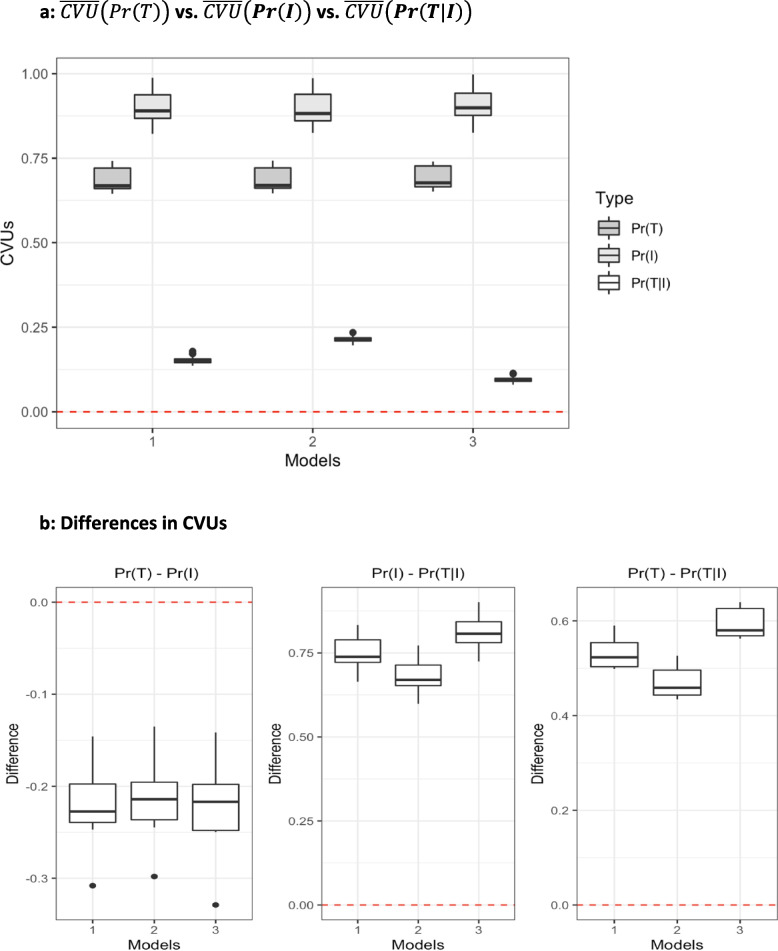
**a** $\overline{CVU }\left(Pr\left(T\right)\right)$ **vs.** $\overline{CVU }\left({\varvec{P}}{\varvec{r}}\left({\varvec{I}}\right)\right)$ **vs.** $\overline{CVU }\left({\varvec{P}}{\varvec{r}}\left({\varvec{T}}|{\varvec{I}}\right)\right)$. **b** Differences in CVUs

In the following section we present the results for hypothesis testing for the three hypotheses presented in Eqs. 3a- 3c. We begin by demonstrating that the regional variations in the probability of treatment, in the probability of initial visit and probability of treatment given an initial visit are statistically significant from zero. This is demonstrated by the box plot of $\overline{CVU }\left(Pr(T)\right)$, $\overline{CVU }\left(Pr(I)\right)$ and $\overline{CVU }\left(Pr(T|I)\right)$ in Fig. 1a using bootstrapped standard errors. All CVUs are statistically significant, indicating the presence of practice variation between regions. $\overline{CVU }\left(Pr(I)\right)$ is the largest in terms of magnitude, followed by $\overline{CVU }\left(Pr(T)\right)$ and finally $\overline{CVU }\left(Pr(T|I)\right),$ for all model specifications. It is worth noting that regional dispersion in $Pr(T|I)$ is considerably smaller when compared to the other two. On the other hand, different specifications of the model whereby we control for observed patient needs (Model 2) and observed patient needs and preferences (Model 3) lead to only slight difference in CVUs in the first two cases, while Model 3 results in a slightly lower regional dispersion in the case of $\overline{CVU }\left(Pr(T|I)\right)$.

Next, we continue with our hypothesis testing by demonstrating that the difference between the in CVUs of each probability in Eqs. 3a- 3c is statistically significant from zero. In Fig. 1b, we present this in three boxplots each representing one of the three hypotheses in in Eqs. 3a- 3c, respectively. All three indicate statistically significant differences in CVUs. Therefore, we can reject the null hypotheses in all three cases. When testing different model specifications, we observe that the difference is larger for Hypothesis 2 and Hypothesis 3 when all observed patient needs and preference variables are added to the model. As seen above, controlling for patient needs and preference variables did not alter $\overline{CVU }\left(Pr(T)\right)$ and $\overline{CVU }\left(Pr(I)\right)$, but led to a slight decrease in the $\overline{CVU }\left(Pr(T|I)\right)$. Hence, the difference between difference between $\overline{CVU }\left(Pr(T)\right)$ and $\overline{CVU }\left(Pr(T|I)\right)$ (and respectively between $\overline{CVU }\left(Pr(I)\right)$ and $\overline{CVU }\left(Pr(T|I)\right)$ is also larger.

## Discussion

Previous scientific research on medical practice variation in treatments has almost exclusively evaluated the magnitude of variation in care at the end-stage of the patient journey, usually at the specialists’ office [9]. Even though several papers have mentioned the need to assess the share of variation originating from events that occur before the patient reaches the specialists office (e.g. patients’ preferences to seek help, GP’s willingness to refer the patient to the specialist) which could explain a significant share of the variation, to the best of our knowledge no empirical research has addressed the issue.

In the present paper, we seek to establish a methodology to isolate the effect of events that occur at each step of the care process. We illustrate our method using care for varicose veins in the Dutch health care system and focus on just two events that require minimal patient journey data (patient’s access to an initial visit with the specialist and receiving treatment given an initial visit having occurred). We calculate the probabilities of treatment, initial visit and of treatment *conditional* on an initial visit. We use three model specifications for our regressions: in Model 1, we control only for patient’s postal code, in Model 2 we add observed variables indicating patient needs (age, obesity, childbirth), and in Model 3 we add observed variables indicating patient preferences (patient’s sex, income, SES, region’s level of urbanization). Next, we calculate the coefficient of variation (CVU), or the measure of dispersion between regions to quantify the amount of variation observed.

We test whether the CVUs of the probability of receiving treatment, the probability of having an initial visit, and conversely, the probability of receiving treatment conditional on an initial visit, are statistically different. If the magnitude of dispersion is larger at the stage of the initial visit, then we can confirm that practice variation was reduced by the specialists in the clinics. And vice-versa, if we find that coefficient of variation was lower at the stage of an initial visit than at the stage of the treatment, we can conclude that the clinics amplified practice variation further.

Our results demonstrate that the CVU, or the measure of dispersion, in the regional probability of an initial visit was significantly larger (0.87–0.96) than at the point of treatment both conditional (0.14–0.25) and unconditional on an initial visit (0.65–0.74), suggesting that practice variation was present before the patient reached the specialist. This suggests that at least part of the variation stems from decisions made before the patient reaches the specialist. Furthermore, when comparing the dispersion in the probability of treatment and the dispersion in the probability of treatment given an initial visit, we notice that the latter is smaller. This indicates that specialists were able to reduce the variation in treatment acting as a ‘gate-keeper’, a role that is traditionally associated with primary care.

We observed that the specialist’s decision meaningfully diminished the amount of regional variation. However, the variation did not entirely disappear at any stage, even after we controlled for patient needs and patient preferences. Our data did not allow us to correct for all need factors and probably only a small share of differences in preferences. Hence, a significant share of the treatment variation remained ‘unexplained’. Factors such as religion, ethnicity, education, household income, previous health care use, proximity to the closest GP or specialist, and the financial health of the provider may also play an important role. Some of these variables (e.g. income, SES) were substituted with regional averages, which may limit their explanatory power in our regressions. Similarly, although our analysis demonstrated the presence of regional dispersion at the initial visit stage, this may not be fully attributed to decisions made by the general practitioner. We expect that much of the observed variations may find their origin in variations in the patients’ willingness to seek care. Primary care physicians are assumed to act as a gatekeeper to guard against the clinically unnecessary use of the collectively insured care. As there is no evidence of underuse from a clinical perspective, we assume that much of the variation in use results from non-clinical (i.e. esthetical) demands for care, which suggests that the GP’s gatekeeping role might have been ignored. It remains unclear to what extend regional variations in willingness to refer are the cause for variation. Our administrative dataset did not allow us to utilize expressed patient needs and preferences variables such that clinical data or survey data would have allowed. Therefore, our needs and preference variables were only proxies with presumably high level of correlation with the actual needs and preferences of the patient. If we assume that the proxy variables used in our model are adequately correlated with the true expressed variables, they can be used to indicate the direction of correlation with some ‘random’ measurement error. Therefore, using proxies, although imperfect, would lead to attenuation bias, and therefore an underestimation of the true effect. If, however, particular preferences did not correlate with our proxy measures, then our analysis could suffer from unmeasured confounding and biased results in an unpredictable direction. It would however not affect the validity of the method that we have introduced.

Our method can be extended by disaggregating and decomposing the results. As the average contribution of both steps may not be equal in every region, analysis per region may clarify the contribution of region-specific referral patterns and individual clinics in our findings. While the point estimates of contributions by region may be interesting, this would require additional attention with respect to the computation of confidence intervals, as bootstrapping would occur on a regional level. If the number of observations become low and confidence intervals wide, then our frequentist approach might result in unstable estimates. Shrinkage approaches, like empirical Bayes, might be appropriate but would require further calculations. They were, therefore, beyond the scope of this study.

In addition, our method could be used to decompose the overall contribution to variation originating from each step of the patient journey when using a model that is linear in the parameters (e.g. ANOVA or linear regression). For such decompositions one may use the law of total covariance, which states that if X,Y,Z are random variables then:

*cov*(*X*,*Y*) = *E*(*cov*(*X*,*Y*∣*Z*) + *cov*(*E*(*X*∣*Z*),*E*(*Y*∣*Z*)).

For example, one could use the probabilities of treatment, initial visit, and treatment conditional on initial visits as a continuous dependent variable and decompose using to the above equality. Likewise, the contribution of both stages to the total can be computed using non-linear (multiplicative) decomposition techniques, like the Oaxaca-decomposition. Such decompositions are complex to interpret, and therefore require future exploration.

## Conclusion

In the present paper we offer a methodology for attributing practice variation by patient need, patient preferences, and patient referral along different stages of the patient journey. Our application demonstrates that regional variation in the treatment of varicose veins in the Netherlands is mostly driven by patient and GP decisions leading up to the initial visit to the specialist office. Based on our findings we conclude that any attempt to detect deviations from the norm in the treatment styles of healthcare providers, researchers must take into account the decisions of the patient to seek care and gatekeeping GPs or other referring physicians before reaching the specialist office. The method is easily adaptable to other diagnoses, health care settings and lower levels of aggregation.

## Data Availability

The data that support the findings of this study are available from Zilveren Kruis, but restrictions apply to the availability of these data, which were used under license for the current study, and so are not publicly available. Data are however available from the corresponding author upon reasonable request and with permission of Zilveren Kruis.

## Footnotes

1 Coefficient of variation (CVU) is the ratio of the measure of variability, usually the standard deviation divided by the average about which the variation occurs. It is commonly used statistic in economics, physics, and engineering. (Reed, Lynn, & Meade, 2002), (Lewis & Rao, 2015),(Tateno & Robinson, 2006).

2 We resample our population of plan-holders 1000 times and calculate the difference between CVU(Pr(I)) and CVU(Pr(T|I)) each time, then we infer the distribution of said statistics to determine confidence interval.

3 The impact of parametrization is usually negligible. If not, for example because of a deviating error distribution, it will affect both crude and corrected results similarly and facilitate comparison. These probabilities are very close to the crude unmodeled probabilities, as our regression model is near mean preserving, and would be identical in when using ordinary least squares. While regressions are not needed for this step, they facilitate programming and direct comparison.
